# High‐sugar diet intake, physical activity, and gut microbiota crosstalk: Implications for obesity in rats

**DOI:** 10.1002/fsn3.1842

**Published:** 2020-09-09

**Authors:** Viviano Gomes de Oliveira Neves, Daiane Teixeira de Oliveira, Deborah Campos Oliveira, Luiza Oliveira Perucci, Talita Adriana Pereira dos Santos, Isabela da Costa Fernandes, Graziele Galdino de Sousa, Natália Rocha Barboza, Renata Guerra‐Sá

**Affiliations:** ^1^ Núcleo de Pesquisas em Ciências Biológicas Universidade Federal de Ouro Preto Ouro Preto Brazil; ^2^ Programa de Pós‐graduação em Ciências Farmacêuticas Escola de Farmácia Universidade Federal de Ouro Preto Ouro Preto Brazil

**Keywords:** high‐sugar diet, microbiota, obesity, physical activity, swimming training

## Abstract

This study aims to evaluate the effect of long‐term high‐sugar diet (HSD) intake and regular physical activity on gut microbiota as well as its health impact. Weaned male Wistar rats were fed with standard chow diet (SSD) or HSD ad libitum and subjected or not to regular swimming training with a workload (2% of body weight) for 15 weeks. Feces samples were used on microbiome analysis using 16S rRNA amplicon sequencing. HSD increased body mass, adipose cushions, and the serum levels of triglycerides and VLDL, also changed the bacteria taxons associated with metabolic disorders (increase taxons belonging to Proteobacteria phylum and decrease *Pediococcus* genus); the swim training reverted these changes. SSD intake increased the abundance of bacteria associated with metabolization of dietary fiber. Training in association with SSD consumption beneficially modulated the microbiota, increasing the Bacteroidetes, Bacteroidaceae, Porphyromonadaceae, *Parabacteroides*, and Lactobacillaceae, and decreasing the Firmicute/Bacteroidetes ratio; training was not able to maintain this profile in animals SHD‐fed. Physical training modulates the gut microbiota reversing the obesogenic response caused by SHD. However, training itself is not efficient for up‐regulating the probiotic bacteria in comparison to its association with a balanced diet.

## Graphical abstract

In the present study, we showed that among sedentary animals, chronic consumption of the high‐sugar diet for 15 weeks led to metabolic dysfunctions mediated by an increase in triglycerides and VLDL levels, body mass gain and the adiposity index increase. Such findings were associated with taxonomic level changes of the Bacteria kingdom. More specifically, the consumption of the high‐sugar diet led to an increase of specific subclasses belonging to the Proteobacteria phylum: Enterobacteriales order, Enterobacteriaceae family, Helicobacteriaceae family and Escherichia genus. The training was able to reverse the obesogenic effect caused by the high‐sugar diet, and such event was accompanied by the reduction of the abundance rate of taxonomic levels, which were up‐regulated by the high‐sugar diet (Enterobacteriales order, Enterobacteriaceae and Helicobacteriaceae families, and Escherichia and Pediococcus genus).

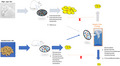

## 1 INTRODUCTION

The consumption of simple carbohydrates, mainly of processed products with high dose of added sugars, has increased substantially in the last decades (Rippe & Angelopoulos, 2016; Yudkin, 1972). The increase of per capita sugar consumption has been pointed out in numerous studies as one of the causes of the development of metabolic diseases such as obesity and diabetes (Kelishadi, Mansourian, & Heidari‐Beni, 2014; Malik, Schulze, & Hu, 2006). In fact, studies show that the consumption of diets with high‐sugar content leads to the development of obesity and metabolic disorders characteristic of human metabolic syndrome, irrespective of caloric intake (Oliveira et al., 2020; Pinto et al., 2016; Queiroz et al., 2014; Sousa et al., 2018).

The benefits of physical activity have been reported since the 1950s, mainly in the decrease of mortality rate caused by chronic diseases (Espeland et al., 2016). Physical training has enormous benefits for human health and studies show that exercise combined with a restricted diet of low‐calorie consumption can be effective in weight loss (Washburn et al., 2014). This is mainly explained by an energy demand that induces greater fatty acid oxidation, and also a higher production of hormones related to metabolic control (Washburn et al., 2014). Aiming to understand the effects of regular physical exercise on a high‐sugar diet, our research group showed in a previous study that the swim training on a Wistar rat model was able to significantly reduce adiposity index in animals due to the consumption of high‐sugar diet (Queiroz et al., 2012).

In recent years, after the advent of metagenome techniques (Amrane & Lagier, 2018; Jandhyala, Talukdar, Subramanyam, Vuyyuru, & Nageshwar Reddy, 2015), it has been showed that the microbiota composition plays a key role in host health since it is capable of producing metabolites such as vitamins (LeBlanc et al., 2013), short‐chain fatty acids (Besten et al., 2013), interfering in many biological processes in the host, such as energy balance (Arora & Sharma, 2011; Cani & Delzenne, 2007; Musso, Gambino, & Cassader, 2010). As reviewed by Conterno et al. and Davis, the human gastrointestinal microbiota differs in composition between eutrophic and obese individuals (Conterno, Fava, Viola, & Tuohy, 2011; Davis, 2018). The microbiota composition is shaped by some factors such as diet (David et al., 2014; Prescott, 2017) and physical activity (Campbell & Wisniewski, 2017; Denou, Marcinko, Surette, Steinberg, & Schertzer, 2016).

Based on previous data from our research group and the new findings about the influence of the microbiota on host health, we focused on the evaluation of whether the swimming training with a workload could reverse the effects of chronic feed high‐sugar diet by modulating the bowel microbiota and, as consequence, improving the energetic metabolism of the host.

## 2 EXPERIMENTAL SECTION

### 2.1 Animals, diet, and physical training

Thirty‐two newly weaned (with 28 old days) male Wistar rats (56.93 ± 5.2 g) were used in this study. All experimental procedures were performed according to the Brazilian guidelines of animal experimentation of the National Council for the Control of Animal Experimentation (CONCEA) and approved by the Ethics Committee in the Use of Animals of the Federal University of Ouro Preto (UFOP) (protocol number 26/2016). The animals were kept in collective cages under controlled light conditions (light and dark cycle of 12:12 hr) and room temperature of 24 ± 2°C with ad libitum access to water and food.

Wistar rats were randomly divided into four groups: sedentary standard chow diet (SSD, *n* = 8), trained standard chow diet (TSD, *n* = 8), sedentary high‐sugar diet (SHD, *n* = 8), and trained high‐sugar diet (THD, *n* = 8). The animals of SSD and TSD groups were fed with a standard rat chow (Nuvilab CR1®, Colombo, Brazil) composed of 57.16% of carbohydrate (being 0% added sugar). The animals of SHD and THD groups were fed with high‐sugar diet composed of 66.86% carbohydrate (being 36.32% added sugar). The nutritional composition table of the diets has been previously published by the literature (Oliveira et al., 2020). All groups were fed with their respective diets along 15 weeks.

The training groups (TSD and THD) underwent regular swim training. The physical activity protocol was performed in a pool (150 cm × 100 cm × 60 cm wide) containing preheated water at 31ºC ± 2ºC. An adaptation to the aquatic environment and swim training was performed before the regular physical training as follow: On the first training day, the animals swam for 15 min, and in the next days, it was increased 15 min per day, reaching a maximum of 60 min on the fourth day of the adaptation week. In the second week, the animals completed swim sessions of 60 min for five days. After this period, from the third week onwards, the animals were submitted to 60‐min swimming sessions daily with a workload of 2% of their body weight for five days per week. The weight loads were attached on their tails. After each swimming session, the animals were dried to avoid decreases in their body temperature. This experimental assay lasted 15 weeks in total.

### 2.2 Sample collection and metabolic parameters analyses

On the 15th experimental week, the animals (five animals per group) were allocated in the metabolic cage for a period of 24 hr. Feces samples were collected and immediately stored in ultra‐low freezer at −80°C for further DNA extraction and analysis. At the end of the trial period, 24 hr after completion of the physical training protocol, the animals were submitted to fasted state and euthanized by inhalation of carbon dioxide (100% of carbon dioxide gas at a gradual‐fill rate of 20%–30% of the chamber volume per minute). Fat pads (retroperitoneal, epididymal, and inguinal) were dissected and weighed to evaluate the Adipose Index using the following equation: (Fat pad weight (g)/Body weight (g) × 100) (Taylor & Phillips, 1996). Their blood samples were also collected and centrifuged 800 rpm for 15 min. Then, following biochemistry makers were measured in the serum: cholesterol, creatinine, glucose, low‐density lipoprotein (LDL), high‐density lipoprotein (HDL), urea, triglycerides, and very low‐density lipoprotein (VLDL). All biochemistry parameters were measured using Bioclin/Quiabasa kits (Belo Horizonte, MG, Brazil), according to the manufacturer's protocols.

### 2.3 Fecal microbiota analysis

#### 2.3.1 DNA fecal extraction

The feces were kept frozen at −80°C until the extraction process. DNA extraction assay was performed according to the Fecal DNA extraction International Human Microbiome Standards (IHMS) Protocol H (http://www.microbiome-standards.org/). However, an adaptation of this protocol was made before step 16: Chloroform (1:1) was added in supernatants, mixed for 5 min, and then centrifuged at 12,000 rpm for 10 min. The supernatant was collected without intermediates layer contaminants. After this step, all the next procedures were performed exactly as described in the IHMS protocol. The DNA was quantified using the Qubit 3.0 fluorometer (Thermofisher Scientific). The protein contamination was evaluated by the A260/A280 ratio using nanodrope (Thermofisher Scientific), and DNA integrity was evaluated by 0.6*%* agarose gel electrophoresis.

##### 16S ribosomal RNA gene sequencing and analysis

The composition of gut microbiome was assessed by prokaryotic 16S ribosomal RNA (rRNA) gene sequencing of the DNA extracted from the feces. The V4 region of the 16S rRNA gene was amplified with the Foward primer 515 (GTGCCAGCMGCCGCGGTAA) and Reverse primer 806 (TAATCTWTGGGVHCATCAGG) (Caporaso et al., 2011), following the manufacturer's instructions 16S RNA Illumina. A pair‐end library was constructed using Nextera^®^ DNA Library, and high‐throughput sequencing was performed on Illumina MiSeq, kit V2 500 Cycle (Illumina, San Diego, CA, USA).

Amplicon 16S rRNA gene was submitted to the Metagenomics Analysis Server (MG‐RAST) pipeline (http://metagenomics.anl.gov) where the data have gone through a pipeline to remove DNA artifacts derived from the host and duplicate sequencing reads (Meyer et al., 2008). For taxonomic analysis, BlastN from the different ribosomal RNA libraries was used against the M5RNA nonredundant ribosomal gene databases, which choose greengene bank (2006) for validation abundance level for all sample group and using the classification of the best hits, it occurred according to the following criteria maximum e‐value 1–5, minimum identity 90%, and minimum alignment length 15 bp. Using MG‐RAST taxonomic tools (http://tools.metagenomics.anl.gov/), it was possible to evaluate the main taxons at the class level with the representation in the Cytoscape graphic (Franz et al., 2016). For a specific analysis of abundance in the different taxons, levels were performed through a bioinformatics analysis of 16S rRNA gene amplicons at the online portal Illumina (http://www.illumina.com/) to access Basespace at https://basespace.illumina.com/ home/index, and the 16S metagenomics Basespace application was applied to the data. 16S rRNA reads were performed with version of RDP Naive Bayes taxonomic classification algorithm. FASTQ sequences were uploaded to Basespace, and the 16S metagenomics application was executed. After assembling, full‐length sequences from paired ends were referenced against the Illumina‐curated version of Greengenes data base (May 2013). In the same application, 16S metagenomic Base Space it was possible to obtain the species estimate through the Shannon diversity calculation (16S MetagenomicsApp User Guide). To calculate the abundance of microorganisms, the following formula was applied: Abundance = (Specific reads identified in taxonomic level/total number of reads)*100.

### 2.4 Statistical analyses

Statistical analyses were realized by package software GraphPad Prism, version 6.01 (GraphPad Software, San Diego, CA, USA). All the analyses used the two‐way ANOVA test followed Bonferroni's post hoc test, considering *p*‐value <.05 as statistically significant results. Data were presented as mean ± standard deviation.

## 3 RESULTS

### 3.1 Regular swim training reverses the obesogenic effect induced by high‐sugar diet

Table 1 presents the results of the biochemical analysis from different serum metabolites of the experimental groups. According to the results, serum triglyceride and VLDL levels were significantly affected by diet (*p* < .05) and by regular physical activity with workload (*p* < .05). More specifically, the consumption of the high‐sugar diet for 15 weeks induced a significant increase of triglycerides and VLDL in the SHD group compared to the SSD control and the TSD groups. However, regular physical activity with workload was efficiently reversing such events, that is, restorated the serum levels of these metabolites to compatible levels of animals from the SSD group.

**TABLE 1:** Biochemistry parameter of Wistar rats submitted the experimental groups: sedentary standard chow diet (SSD), trained standard chow diet (TSD), sedentary high‐sugar diet (SHD) and trained high‐sugar diet (THD) for 15 weeks

Parameter	Groups *M* ± *SD*				*p*‐value		
	SSD	TSD	SHD	THD	Diet Effect	Training Effect	Interaction
Cholesterol mg/dl	83.0 ± 11.0	84.0 ± 16.0	75.0 ± 14.0	77.0 ± 21.0	>0.99	>0.99	>0.99
Creatinin mg/dl	0.74 ± 0.18	0.64 ± 0.05	0.77 ± 0.05	0.88 ± 0.11	>0.99	0.86	>0.99
Glucose mg/dl	119.0 ± 15.0	122.0 ± 28.0	132.0 ± 11.0	126.0 ± 18.0	>0.99	>0.99	>0.99
HDL mg/dl	23.0 ± 2.5	26.0 ± 2.0	23.5 ± 3.0	26.0 ± 5.0	>0.99	0.82	>0.99
LDL mg/dl	40.0 ± 6.0	41.0 ± 14.0	25.0 ± 11.0	32.0 ± 17.0	0.15	>0.99	>0.99
Triglycerides mg/dl	96.0 ± 33.0	83.0 ± 16.0	151.0 ± 42.0^a^, ^c^	95.0 ± 28.0^b^	<0.05	<0.05	>0.99
VLDL mg/dl	19.0 ± 7.0	16.5 ± 3.0	30.0 ± 8.0^a^, ^c^	19.0 ± 6.0^b^	<0.05	<0.05	>0.99
Urea mg/dl	56.0 ± 7.0	59.0 ± 17.0	48.0 ± 6.0	50.0 ± 19.0	0.75	>0.99	>0.99

Note

Data are expressed as the mean ± standard deviation (*M* ± *SD*). Data tested using Two‐Way ANOVA Test with Bonferroni's post‐test correction. *p* < .005 was considered statistically significant.

Abbreviations: SHD, sedentary high‐sugar diet; SSD, sedentary standard chow diet; THD, trained high‐sugar diet; TSD, trained standard chow diet.

^a^ Denotes significant difference in comparison to the SSD group.

^b^ denotes significant difference in comparison to the SHD group.

^c^ denotes significant difference in comparison to the TSD group. *N*: 8 animals per group.

The biometric profile data are presented in Table 2. The consumption of high‐sugar diet led to an increase of the animals’ body mass (*p* < .05) and mass of white adipose tissue deposits, epididymal (*p* < .01), inguinal (*p* < .05) and retroperitoneal (*p* < .05) adipose tissue, and also to an increased adiposity index (*p* < .01) in the SHD group compared to the SSD and TSD groups. Furthermore, regular physical activity with workload in animals fed with a high‐sugar diet reverted these parameters in the THD group.

**TABLE 2:** Biometric parameter of Wistar rats submitted the experimental groups: sedentary standard chow diet (SSD), trained standard chow diet (TSD), sedentary high‐sugar diet (SHD) and trained high‐sugar diet (THD) for 15 weeks

Parameter	Groups *M* ± *SD*				*p*‐value		
	SSD	TSD	SHD	THD	Diet Effect	Training Effect	Interaction
Body mass (g)	422.0 ± 36.0	362 ± 23.6	480 ± 33.0^a^, ^c^	417.0 ± 48^b^	<0.05	<0.05	0.055
Naso‐anal length (cm)	22.8 ± 1.36	22.4 ± 0.9	24.0 ± 1.2	22.8 ± 1.4	>0.05	>0.05	0.055
Inguinal (g)	10.0 ± 3.0	6.0 ± 2.0	15.0 ± 4.0^a^, ^c^	9.6 ± 4.0 ^b^	<0.05	<0.01	0.49
Epididymal (g)	6.5 ± 2.0	3.5 ± 1.3	13.0 ± 4.0^a^, ^c^	7.5 ± 3.6^b^	<0.01	<0.01	0.23
Retroperitoneal (g)	6.5 ± 2.6	3.13 ± 2.5	13.0 ± 4.5^a^, ^c^	6.9 ± 4.6^b^	<0.05	<0.05	0.36
Adiposity Index	5.6 ± 1.5	3.7 ± 1.5	8.8 ± 2.0^a^, ^c^	5.8 ± 2.1^b^	<0.01	<0.01	0.48

Note

Data are expressed as the mean ± standard deviation (*M* ± *SD*). Data tested using Two‐Way ANOVA Test with Bonferroni's post‐test correction. *p* < .005 was considered statistically significant.

Abbreviations: SHD, sedentary high‐sugar diet; SSD, sedentary standard chow diet; THD, trained high‐sugar diet; TSD, trained standard chow diet.

^a^ Denotes significant difference in comparison to the SSD group.

^b^ denotes significant difference in comparison to the SHD group.

^c^ denotes significant difference in comparison to the TSD group. *N*: 8 animals per group.

### 3.2 Analysis of the fecal microbiome

**FIGURE 1:**
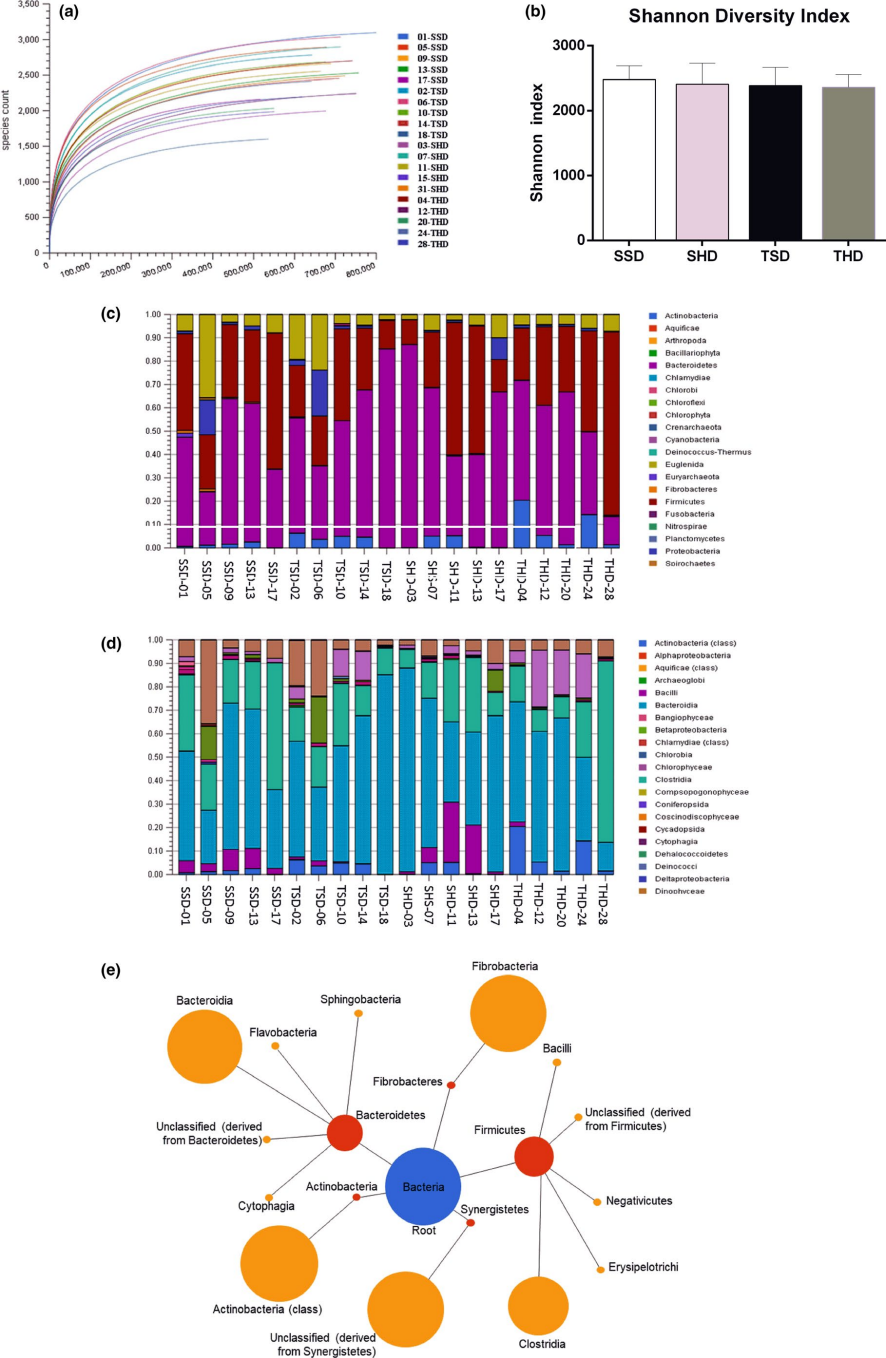
Analysis of fecal microbiome samples of Wistar rats. (a) Rarefaction curves for 16S rDNA sequences. The x‐axis represents the number of sequences generated and the y‐axis represents the richness of species detected. Data generated by MG‐RAST. The correspondence between the curves and samples is shown at the bottom right of the legend. (b) Shannon diversity index. Data tested using a Two‐Way ANOVA Test with Bonferroni's post‐test correction. Data generated by 16S Base Space Illumina App. (c) Fecal microbiota composition among experimental groups at phylum level. Data generated by MG‐RAST. (d) Fecal microbiota composition among experimental groups at class level. Data generated by MG‐RAST. (e) The main bacterial groups identified at phylum and class levels among the experimental groups. The red node represents a phylum and class respectively, and the sizes of the nodes are proportional to the average relative taxonomic abundances. Data generated by MG‐RAST. N: 5 animals per group. SSD, sedentary standard chow diet; TSD, trained standard chow diet; SHD, sedentary high‐sugar diet; THD, trained high‐sugar diet

The effect of high‐sugar diet and regular swim training in the gut microbiota compositions of rats was determined through 16S rRNA sequencing. The sequences obtained were mapped on the green gene database. On average, readings of the 16S rRNA sequence for each sample (~580 thousand) were obtained by 16S Basespace Illumina App. As observed in the rarefaction curves (Figure 1a) (and their tendency to reach the saturated plateau), the readings obtained provided sufficient sequencing depth for taxonomic identifications analysis. The absence of changes in the Shannon's diversity index points to homogeneity in the species estimate of the fecal microbiome among the experimental groups (Figure 1b; *p* = .86). Fecal microbiota composition among experimental groups at phylum and class levels is shown in Figure 1c,d, respectively. Bacteroides, Firmicutes and, to a lesser extent, Actinobacteria, Fibrobacteres, and Synergistetes were the most abundant phylum identified in the experimental groups on this study (Figure 1e). At class level, the most abundants were as follows: Bacteroidia, Flavobacteria, Sphingobacteria, Cytophagia, Actinobacteria, Fibrobacteria, Clostridia, Erysipelotrichi, Negativecutes, and Bacilli (Figure 1e).

### 3.3 High‐sugar diet and regular swimming training shape microbiome in different taxonomic levels

**FIGURE 2:**
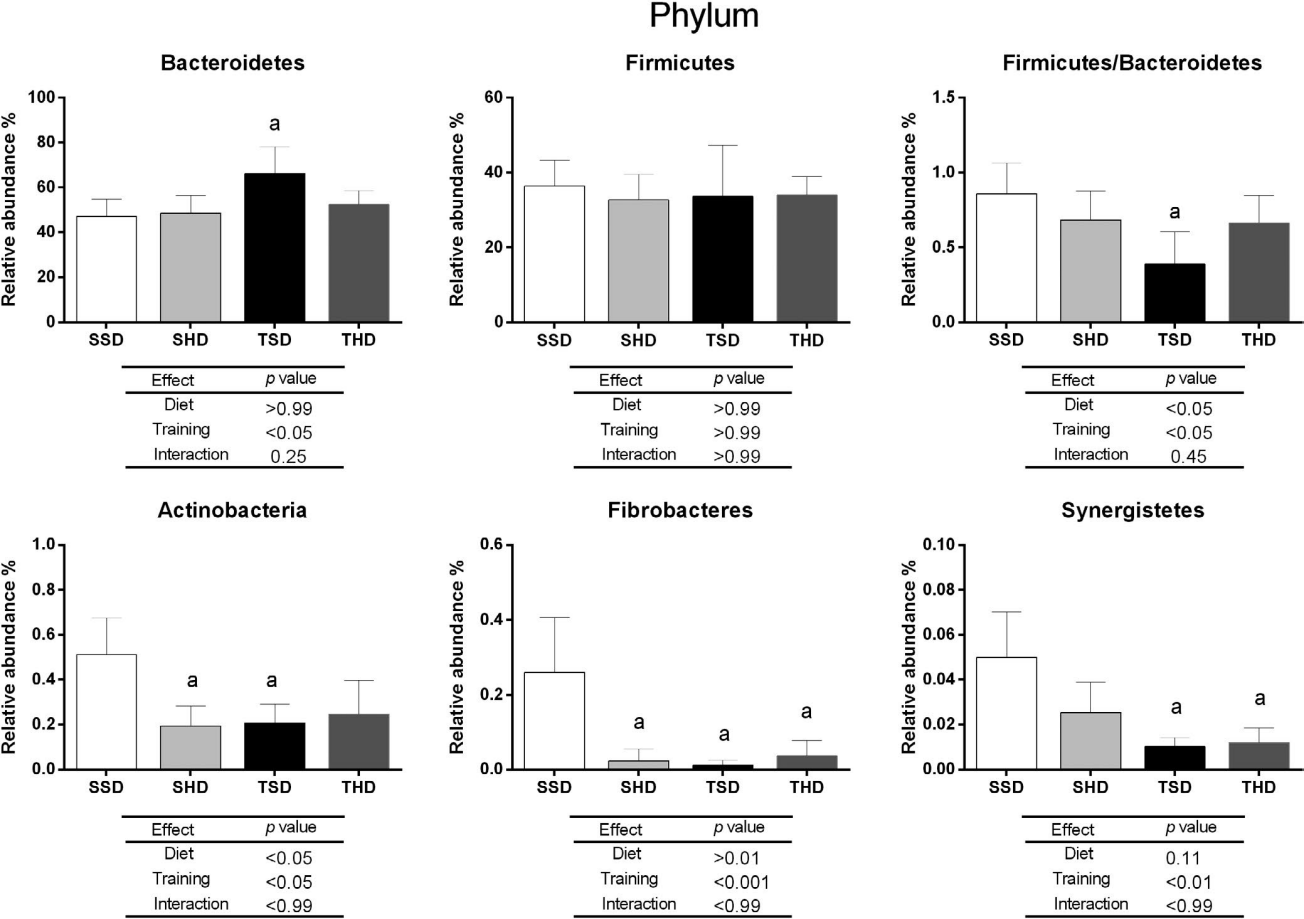
The relative abundance at phylum level of the fecal microbiome of Wistar rats fed by standard chow or high‐sugar diets and submitted or not to swim training. Data are expressed as mean ± standard deviation. Data tested using Two‐Way ANOVA Test with Bonferroni's post‐test correction. *p* < .005 was considered statistically significant. ^a^Denotes significant difference in comparison to the SSD group. Analyses were performed in the 16S Base Space Illuimna App. N: 5 animals per group. SSD, sedentary standard chow diet; TSD, trained standard chow diet; SHD, sedentary high‐sugar diet; THD, trained high‐sugar diet

**FIGURE 3:**
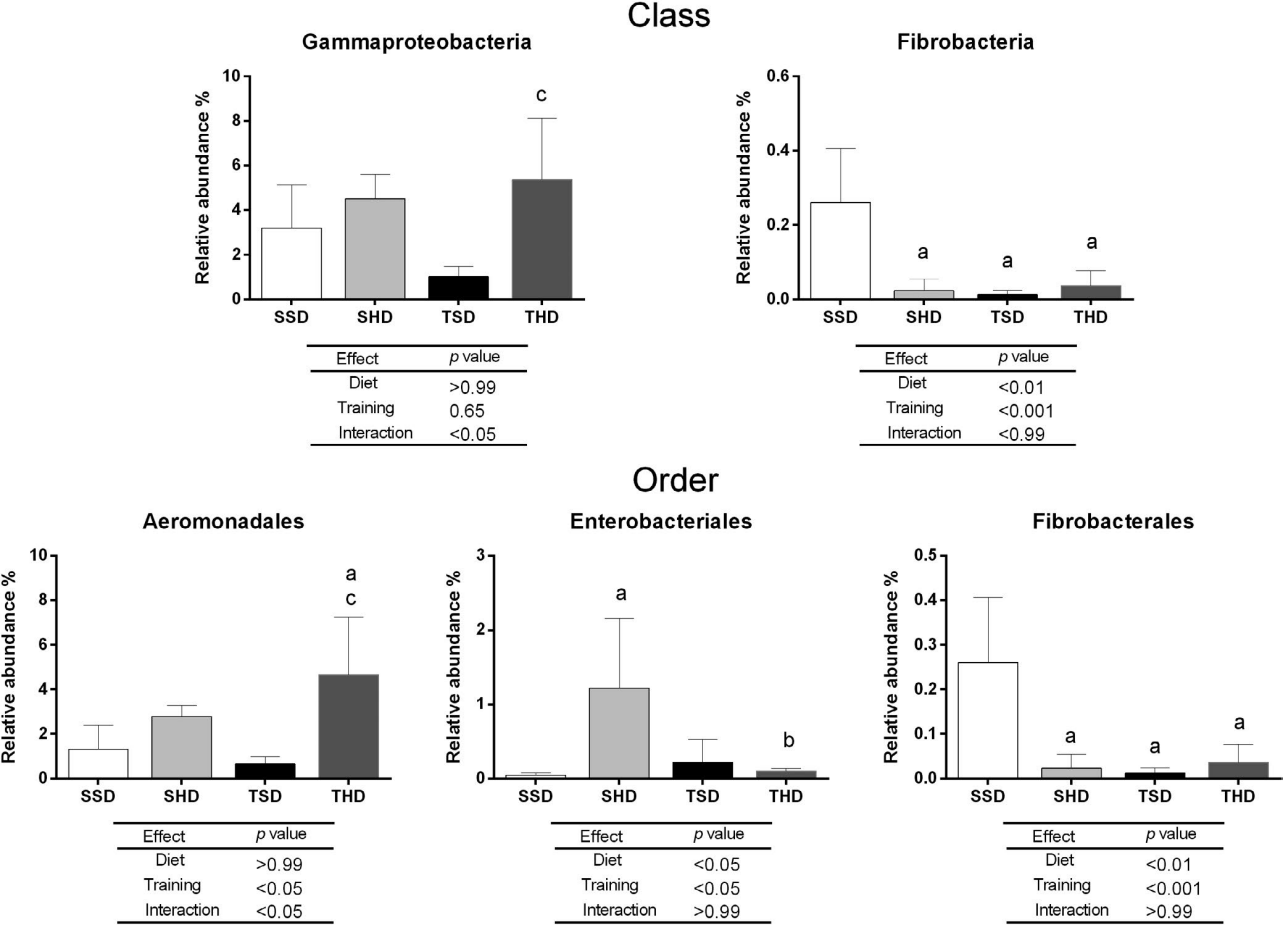
The relative abundance at class and order levels of the fecal microbiome of Wistar rats fed by standard chow or high‐sugar diets and submitted or not to swim training. Data are expressed as mean ± standard deviation. Data tested using Two‐Way ANOVA Test with Bonferroni's post‐test correction. *p* < .005 was considered statistically significant. ^a^Denotes significant difference in comparison to the SSD group, ^b^denotes significant difference in comparison to the SHD group and ^c^denotes significant difference in comparison to the TSD group. Analyses were performed in the 16S Base Space Illumina App. N: 5 animals per group. SSD, sedentary standard chow diet; TSD, trained standard chow diet; SHD, sedentary high‐sugar diet; THD, trained high‐sugar diet

**FIGURE 4:**
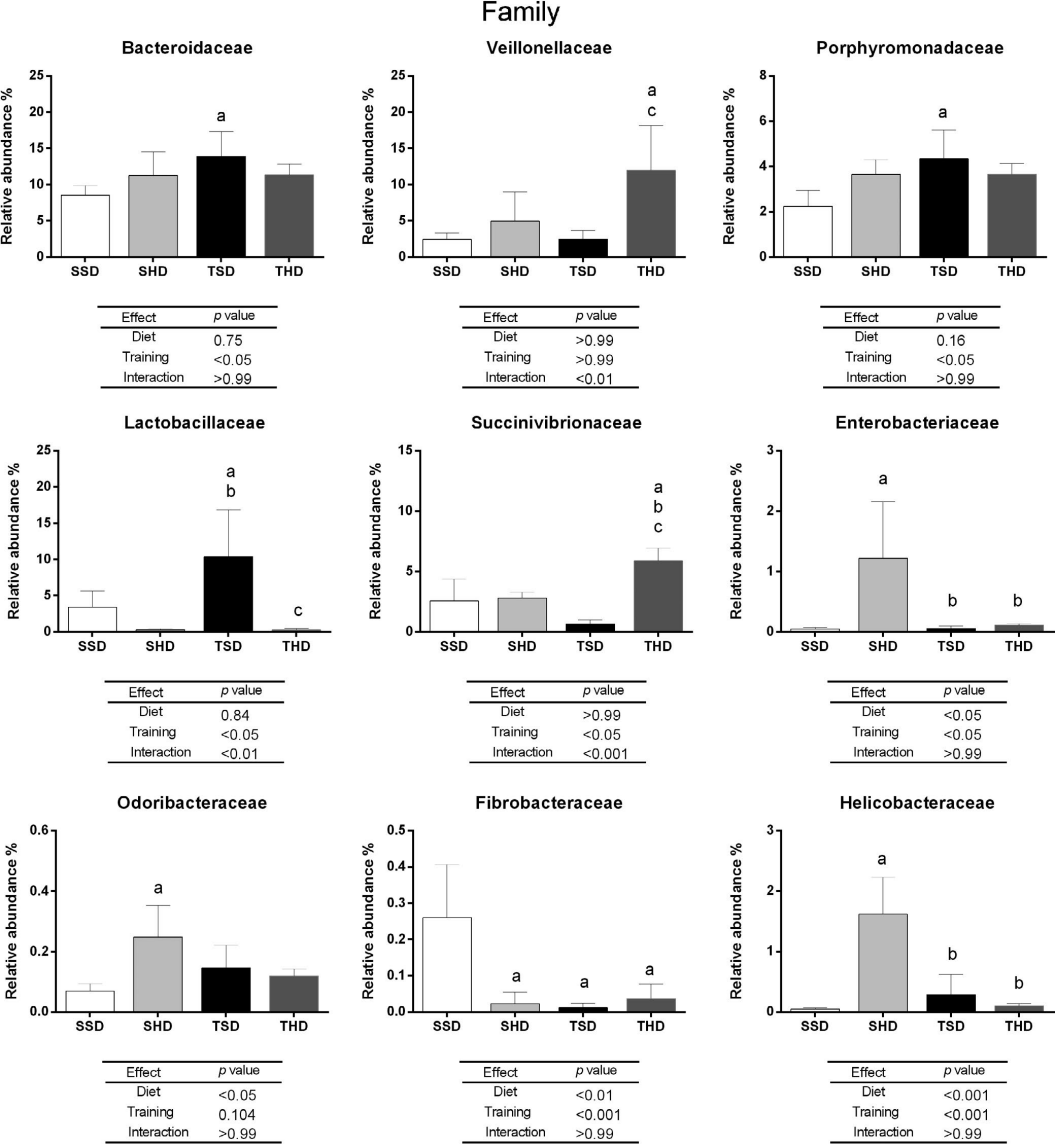
The relative abundance at family level in fecal microbiome of Wistar rats fed by standard chow or high‐sugar diets and submitted or not to swim training. Data are expressed as mean ± standard deviation. Data tested using Two‐Way ANOVA Test with Bonferroni's post‐test correction. *p* < .005 was considered statistically significant. ^a^Denotes significant difference in comparison to the SSD group, ^b^denotes significant difference in comparison to the SHD group, and ^c^denotes significant difference in comparison to the TSD group. Analyses were performed in the 16S Base Space Illumina App. N: 5 animals per group. SSD, sedentary standard chow diet; TSD, trained standard chow diet; SHD, sedentary high‐sugar diet; THD, trained high‐sugar diet

**FIGURE 5:**
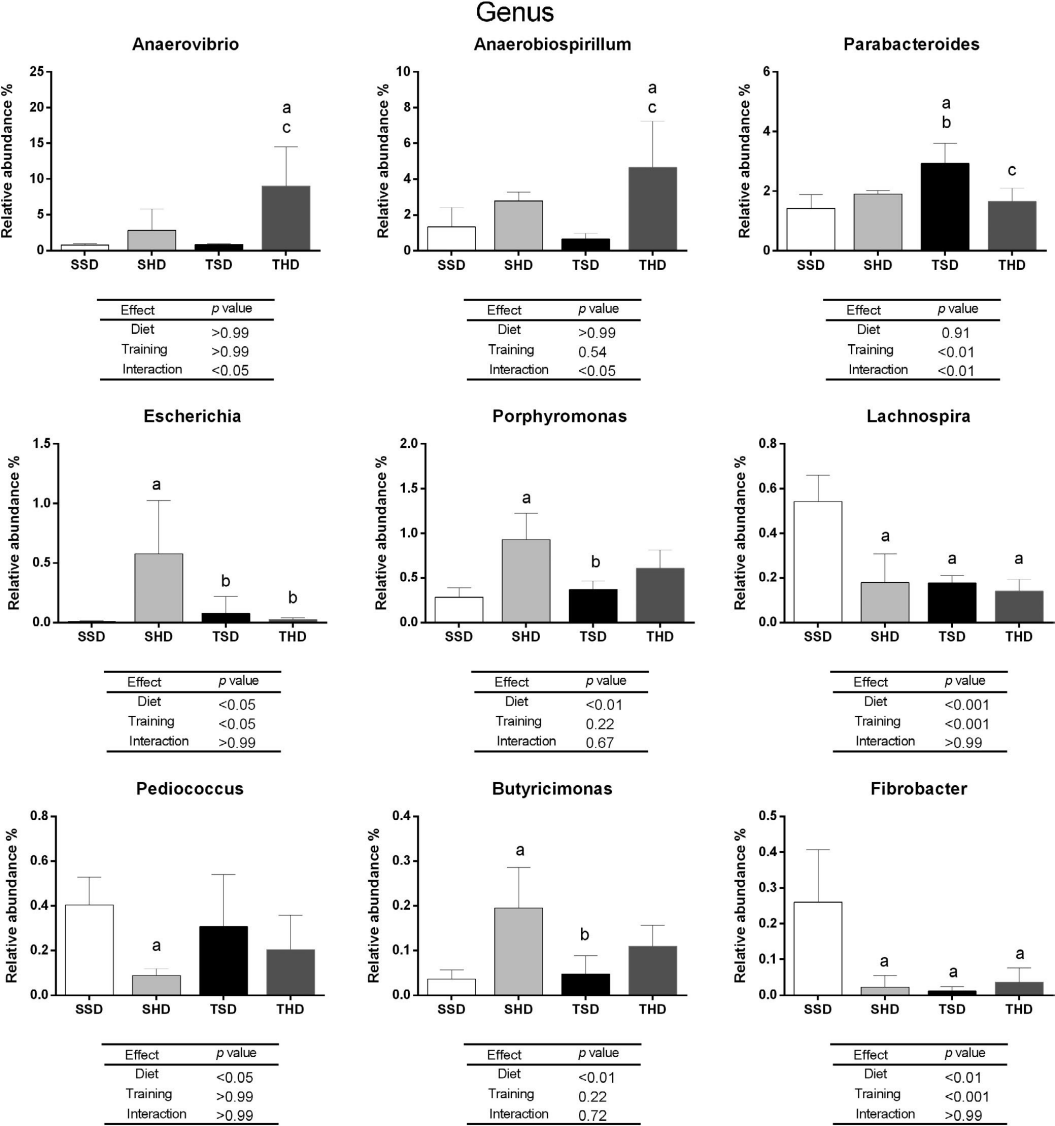
The relative abundance at genus level in fecal microbiome of Wistar rats fed by standard chow or high‐sugar diets and submitted or not by swim training. Data are expressed as mean ± standard deviation. Data tested using Two‐Way ANOVA Test with Bonferroni's post‐test correction. *p* < .005 was considered statistically significant. ^a^Denotes significant difference in comparison to the SSD group, ^b^denotes significant difference in comparison to the SHD group, and ^c^denotes significant difference in comparison to the TSD group. Analyses were performed in the 16S Base Space Illumina App. N: 5 animals per group. SSD, sedentary standard chow diet; TSD, trained standard chow diet; SHD, sedentary high‐sugar diet; THD, trained high‐sugar diet

In this study, 59 phylum, 59 class, 114 order, 244 families, and 610 genus were identified. Nevertheless, only a few taxonomic levels have been significantly changed. The significant changes in relative abundance of distinct taxonomic levels between the experimental groups in the study are represented in Figures 2, 3, 4, 5. At phylum level (Figure 2), the high‐sugar diet reduced the abundance of Actinobacteria (*p* < .05) and Fibrobacteres (*p* < .01) in the SHD group compared to the SSD group. In animals fed with standard chow diet, the swim trained was able to increase Bacteroidetes (*p* < .05) and decrease the Synergistetes (*p* < .01), Fibrobacteres (*p* < .001), and Actinobacteria (*p* < .05) abundance and Firmicutes/ Bacteroidetes ratio (*p* < .05) in the TSD group compared to the SSD group.

At the Class level (Figure 3), the abundance of Fibrobacteria was influenced by diet (*p* < .01) and training (*p* < .001). The consumption of high‐sugar diet, similarly to the training, induced a decrease in Fibrobacteria abundance in SHD, THD, and TSD groups compared to the SSD group. In the trained groups, the high‐sugar diet led to an increased Gammaproteobacteria abundance (*p* < .05) in the THD group compared to the TSD group. The interaction between the high‐sugar diet intake and training also led to an increase in Gammaproteobacteria abundance (*p* < .05).

At the order level (Figure 3), the Enterobacteriales and Fibrobacterial abundance were influenced by diet (*p* < .05 and *p* < .01, respectively) and training (*p* < .05 and *p* < .001, respectively). In sedentary animals, the abundance of Enterobacteriales increased in the high‐sugar diet group compared to SSD group (*p* < .05). Nevertheless, swim training in animals fed with high‐sugar diet was able to reduce Enterobacteriales abundance (*p* < .05) at compatible levels to SSD group animals. On the other hand, both high‐sugar diet and training decreased Fibrobacterial abundance in SHD, THD, and TSD groups compared to the SSD group. The interaction between high‐sugar diet and training (*p* < .05) also increased the abundance of Aeromonadales in the THD group compared to the SHD and TSD groups.

At the Family level (Figure 4), the high‐sugar diet consumption by sedentary animals (SHD group) increased Enterobacteriaceae (*p* < .05), Helicobacteraceae (*p* < .001), and Odoribacteraceae (*p* < .05) abundances compared to the SSD group. Although, regular physical activity with workload reduced Enterobacteriaceae (*p* < .05) and Helicobacteraceae (*p* < .001) levels compared to the SHD group, restoring the abundance of these families to those compatible to the control groups. On the other hand, the high‐sugar diet and training decreased Fibrobacteriaceae abundance in the SHD, TSD, and THD groups compared to the SSD group, an effect of diet (*p* < .01) and training (*p* < .001) being observed. In animals fed with standard chow diet, the swim training was capable to increase the abundance of Bacteroidaceae (*p* < .05), Porphyromonadaceae (*p* < .05), Lactobacillaceae (*p* < .05) in TSD group compared to the SSD group. More specifically, the interaction between standard chow diet and training (*p* < .01) led to an increase in Lactobacillaceae family. The interaction between high‐sugar diet and training led to an increase in Veillonellaceae (*p* < .01) abundance in the THD, while the interaction between high‐sugar diet and training also led to an increase in Succinivibrionaceae (*p* < .001) in the THD compared to SSD, TSD, and SHD groups.

At the genus level (Figure 5), in sedentary animals, the high‐sugar diet increased the abundance of *Escherichia* (*p* < .05), *Porphyromonas* (*p* < .01), *Butyricimonas* (*p* < .01), and decreased abundance of *Pediococcus* (*p* < .05). Nevertheless, swim training decreased *Lachnospira* (*p* < .001) and *Fibrobacter* (*p* < .001) and increased *Parabacteroides* (*p* < .001) abundance in the TSD group compared to the SSD group. The interaction between standard chow diet intake and training (*p* < .01) led to a decrease in *Parabacteroides* (*p* < .001) abundance in the THD group compared to the TSD group. Both high‐sugar diet intake and training led to a decrease in *Lachnospira (p* < .001) and *Fibrobacter* (*p* < .01) abundance in the SHS, TSD, and TSH groups compared to the SSD group. Training also led to a decrease in *Escherichia* (*p* < .05) levels in animals of the THD and TSD groups compared to the SHD group. The interaction between high‐sugar diet intake and training led to an increase in *Anaerovibrio* (*p* < .05) and *Anaerobiospirillum* (*p* < .05).

## 4 DISCUSSION

This study revealed that the high‐sugar diet intake and the regular swim training induced dynamic changes in the fecal microbiota of rats. More precisely, we found that obesity induced by high‐sugar diet consumption profoundly modifies the composition of the intestinal microbiota, changing the abundance rate of taxons associated with the development of pathologies such as obesity. However, training was able to reverse the obesogenic effect induced by the high‐sugar diet and restore the abundance rate of these modified taxons to compatible levels of those animals submitted to standard chow diet. Training in association with consumption of the standard chow diet was also beneficial to the gut microbiota, increasing the abundance rate of bacteria that have probiotic effects. However, such effect of training was not observed in animals that consumed the high‐sugar diet. These findings indicate that the regular swim physical exercise could positively act in the gut microbiota of rats, restoring altered patterns due to the high consumption of sugars to normal levels. However, the training itself is not able to induce beneficial adaptations in the microbiota for host health, in comparison to its association with a balanced diet.

In the present study, among sedentary animals, chronic consumption of the high‐sugar diet for 15 weeks led to metabolic dysfunctions (increase in triglycerides and VLDL levels), body mass gain and white adipose tissue and the adiposity index increase. Such findings were associated with taxonomic level changes of the Bacteria kingdom. More specifically, the consumption of the high‐sugar diet led to an increase of specific subclasses belonging to the Proteobacteria phylum: Enterobacteriales order, Enterobacteriaceae family, Helicobacteriaceae family e *Escherichia* genus. As reviewed by Shin et al., in metabolic disorders, the unstable microbial community (dysbiosis) is often associated with an increased prevalence of Proteobacteria (Shin, Whon, & Bae, 2015). Corroborating with our results, Rong et al. (2016) demonstrated that the increase in Helicobacteriaceae abundance in rats submitted to a high‐fat diet was associated with the development of obesity. Furthermore, potential pathogenic intestinal microbes, belonging to the Enterobacteriaceae family (*Shigella* and *Escherichia*), were significantly increased in European children with a typical Western diet (rich in animal proteins, sugar, starch, and fats and low in fiber) compared to children from a rural African village with a predominantly vegetarian diet (low in fat and animal protein and rich in starch, fiber, and vegetable polysaccharides) (Filippo et al., 2010). The Proteobacteria, Enterobacteriaceae, and *Escherichia* abundance were also significantly elevated in the gut microbiota of nonalcoholic steatohepatitis patients (Zhu et al., 2013). As previously published by our research group, consumption of high‐sugar diet, even under isoenergetic conditions, leads to the development of nonalcoholic fatty liver disease (NAFLD) (Oliveira et al., 2020). In the present study, the high‐sugar diet intake also reduced the abundance rate of the *Pediococcus* genus, composed by acid‐lactic bacteria. Animal model studies of obesity induced by a high‐fat diet (Moon, Baik, & Cha, 2014) and clinical trials (Higashikawa et al., 2016) indicate that species belonging to the *Pediococcus* genus exert an anti‐obesity effect (reduction of body mass and fat).

The training was able to reverse the obesogenic effect caused by the high‐sugar diet, such event was accompanied by the reduction of the abundance rate of taxonomic levels, which were up‐regulated by the high‐sugar diet (Enterobacteriales order, Enterobacteriaceae and Helicobacteriaceae families, and *Escherichia* and *Pediococcus* genus). Corroborating to our findings, other authors observed a reduction in the abundance of bacteria related to the phylum Proteobacteria in rats submitted to physical training (high‐intensity racing) (Liu et al., 2015; Petriz et al., 2014).

Interestingly, still inside the phylum Proteobacteria was observed that the interaction of the training with the high‐sugar diet led to an increased abundance of Gammaproteobacteria class and its subclassification Aeromonadales order, Succinivibrionaceae family, and the *Anaerobiospirillum* genus. As well as other taxons belonging to the Proteobacteria phylum, the increase in Gammaproteobacteria class is related to NAFLD (Michail et al., 2015; Zhao et al., 2019). In a choline depletion study, diet supplementation with a high choline content resulted in a drastic reduction in the abundance of Gammaproteobacteria (Spencer et al., 2011). It is known that strenuous and prolonged physical exercise can emphasize several metabolic pathways, increasing the demand for choline as a substrate and resulting in a decrease in the free choline stock (Penry & Manore, 2008). Thus, in the present study, we hypothesized that gut microbiota alterations may be associated with a synergistic effect of training and diet on the decrease of choline levels. Crosstalk between training and high‐sugar diet intake also increased the abundance of the family of Veillonellaceae and its genus *Anaerovibrio*. Is important to emphasize these taxons, as well as Succinivibrionaceae family, are strictly anaerobic and negative gram (Marchandin & Jumas‐Bilak, 2014; Santos & Thompson, 2014). Training might promote the anaerobic environment in the intestine because it acts to reduce the intestinal blood flow due to vasoconstriction of the splanchnic vascular bed for catecholamines liberation and bound on the adrenergic receptors (Granger, Richardson, Kvietys, & Mortillaro, 1980). At rest, more than 90% of the blood flow which is targeted to the intestine is directed to the mucosa (Chou & Grassmick, 1978), showing that this region is most susceptible to change in consequence of reducing intestinal blood flow induced by training. The anaerobic profile in the intestine promoted by the training favored the growth of the taxa of Gammaproteobacteria and Veillonellaceae added by high‐sugar source. In this sense, it was demonstrated that the growth of genus *Anaerobiospirillum* is favored in anaerobic conditions with a source of simple carbohydrates (Lee, Lee, & Chang, 2008). Low O_2_ circulation in the intestine, fermentation, and degradation of sugars by microorganisms can lead to the production of metabolites such as lactate which could be used for the growth of Veillonellaceae family (Ríos‐Covián et al., 2016).

Sedentary animals fed with the standard chow diet showed a greater abundance of taxa related to the response and metabolization of dietary fiber. More specifically, animals in the SSD group showed an increase of Fibrobacteres phylum, and its subclassification Fibrobacteria classes, Fibrobacterales order, Fibrobacteraceae family and *Fibrobacter* genus, compared to those submitted to high‐sugar diet and training. A similar profile was observed on Actinobacteria phylum and at the *Lachnospira* genus. Already the Synergistetes phylum was increased in exclusively by training. This finding might be related to a higher composition of dietary fibers in the standard chow diet compared to the high‐sugar diet (114.27 g/kg of diet vs. 50.77 g/kg of diet) which has high content of added sugars (Oliveira et al., 2020). Studies point possible correlation between fiber consumption and the modulation of the microbiota in the development of obesity and its associated pathologies (Makki, Deehan, Walter, & Bäckhed, 2018). Accordingly, high fiber intake and the modulation of the diversity of intestinal microbiomes are correlated with lower weight gain in humans submitted to a long run training, regardless of caloric intake (Menni et al., 2017). Likewise, in overweight and obese children, high fiber intake selectively altered the intestinal microbiota and significantly reduced body mass and fat percentage (Nicolucci et al., 2017). Consequently, diets with high glycemic index (with high content of simple carbohydrates and with low fiber content) may be positively associated with mass gain regardless positive energy balance, with microbiota shape induced by the diet being a key factor in this process. Regarding training, the lower abundance of these taxons may be explained by an increase of gastrointestinal tract motility through a high production of intestinal peptides and prostaglandin peptides promoted by aerobic exercise (Lira, Vancini, da Silva, & Nouailhetas, 2008). Therefore, our hypothesis is that the process of degradation and fermentation of food nutrients such as fibers remain a shorter time in the intestine, thus contributing to the lower growth of these microorganisms in the bowel of Wistar rats in this experimental model.

In animals that consumed the standard diet, the regular swim training was able to increase the Bacteroidetes phylum, Bacteroidaceae and Porphyromonadaceae families and the *Parabacteroides* genus. A similar profile was also observed on the Lactobacillaceae family. On the contrary, the training led to a decrease in the ratio of Firmicute/Bacteroidetes phyla in the microbiota of these animals. However, it is worth of mentioning that such training effect was not observed in animals fed with a high‐sugar diet. According to published data, there is a relationship between greater abundance of Bacteroidetes in humans and animals with lean phenotypes as well as those who practice regular exercises (Cortez et al., 2018; Cox‐York et al., 2015; Kasai et al., 2015; Tseng & Wu, 2018). Obese and overweight individuals had a higher Firmicutes/Bacteroidetes ratio in their gut microbiota (Kasai et al., 2015). In C57 BL/6 mice, obesity induced by the consumption of a high‐fat diet was associated with a decrease in the Bacteroidetes/Firmicutes ratio, although physical training (treadmill running) was able to increase this ratio (Denou et al., 2016). Studies have also shown that bacteria belonging to the *Parabacteroides* genus had probiotic effects against the development of obesity and metabolic dysfunctions (Tan et al., 2012; Wu et al., 2018). Genus *Parabacteroides* represents a group of anaerobic bacteria (Tan et al., 2012). We hypothesized that the association of anaerobiosis promoted by training in association with a standard diet which owns high fiber source favored an increase in the abundance of *Parabacteroides* in the TSD group. Carlson et. al showed that partially hydrolyzed fibers favor the growth of probiotic bacteria such as *Parabacteroides* (Carlson, Gould, & Slavin, 2016). Thus, in the present study, we hypothesized that gut microbiota alterations may be associated with a synergistic effect of training and diet on the decrease of choline levels. Furthermore, Lactobacillaceae are part of the lactic acid‐producing bacteria group and exert many benefits as those associated with their probiotic effect, inducing lactose intolerance mitigation, immunomodulation, bile acid resistance and bacteriocin production (Turpin, Humblot, Noordine, Thomas, & Guyot, 2012; Wang et al., 2019). Together, our results indicate that the training associated to a balanced diet can positively modulate the healthy gut microbiome. However, training itself could not be sufficient to maintain this profile in face of a poorly balanced/obesogenic diet.

In conclusion, our results indicate that chronic consumption of a high‐sugar diet leads to the development of obesity and dynamic changes in the fecal microbiota in taxons associated with metabolic disorders and that regular swim training is effective in reversing such changes. Regular swim training associated to a balanced diet also induced beneficial adaptations in the microbiota. However, training itself is not able to maintain this profile, given the consuming an obesogenic diet with a high content of added sugars. Therefore, the gut microbiota shape may mediate the well‐known beneficial effects of regular physical exercises associated with a balanced diet to the host health.

## CONFLICT OF INTEREST

All authors declare no conflict of interest.
